# Metagenomes and metagenome-assembled genomes from a sequentially fed anaerobic digester treating solid organic municipal waste

**DOI:** 10.1128/mra.00919-23

**Published:** 2023-12-21

**Authors:** Camilla L. Nesbø, Temesgen M. Fitamo, Hyunwoo Lee, Elizabeth A. Edwards

**Affiliations:** 1 Department of Chemical Engineering and Applied Chemistry, University of Toronto, Toronto, Ontario, Canada; 2 BioZone, University of Toronto, Toronto, Ontario, Canada; California State University San Marcos, San Marcos, California, USA

**Keywords:** anaerobic digester, metagenome, MAG, municipal waste

## Abstract

We present a data set of four metagenomes and 281 metagenome-assembled genomes describing the microbial community of a laboratory-scale high solids anaerobic digester. Our objective was to obtain information on the coding potential of the microbial community and draft genomes of the most abundant organisms in the digester.

## ANNOUNCEMENT

A 50 kg scale high solids anaerobic digester with leachate recycle, known as “Daisy the Digester” described by Guilford et al. (1), was operated at 37°C for 88 weeks. Methane yield was greatly enhanced by the addition of food waste. The microbial community was monitored using 16S rRNA amplicon sequencing (2). Metagenomes from high solids digesters are rare compared to those from liquid, well-mixed anaerobic digesters. We therefore selected four samples for metagenomic shotgun sequencing to get a blueprint of the functional potential of the community. The aim of this study was to obtain the genomes of organisms that emerged as key 16S rRNA gene amplicon sequence variants in correlative analyses (2).

Daisy was fed constant weekly additions of cardboard, boxboard, newsprint, fine paper, and wood chips, the latter as bulking agent (2). The amount of food waste (FW) fed weekly varied from 0 to 509 g FW as chemical oxygen demand (COD; measured using standard methods [3]), up to 29.3% FW on a COD basis. Leachate samples were collected from the drainage lines of each individual leach bed. Leachate sample S62W2 was taken on 7 June 2016 from leach bed serial #62 (S62) at the start of the second week (W2) of incubation, when the feed contained 17.2% FW as COD. Sample S79W4 was taken on 18 October 2016 at 21.9% FW as COD. Samples S80W1 and S80W4 were taken on 4 October 2016 and 25 October 2016 when the feed composition was 29.3% FW as COD.

DNA extractions were performed using the Qiagen DNeasy PowerSoil kit (Qiagen, Carlsbad, CA, USA) as described in detail by Lee et al. (2). DNA from samples S62W2 and S80W1 were fragmented by Covaris (Covaris Inc., Woburn, MA, USA). Adaptors for PCR amplification were ligated after end-repair and A-tailing. The PCR-enriched adapter-ligated DNA fragments were sequenced using Illumina HiSeq with an average insert size of 250 bp generating pair-end read of 150 nt at BGI, Hong Kong (Table 1). The library from sample S79W4 was prepared using the NEBNext Ultra II DNA Library Prep Kit for Illumina (New England BioLabs) with average library insert size of 250 nt and sequenced using Illumina NovaSeq generating pair-end reads of 150 nt at Genome Quebec, Montreal, Canada (Table 1). The DNA library from Sample S80W4 was prepared using the SMRTbell Express Template Prep Kit 2.0 (Pacific Biosciences, Menlo Park, CA, USA) without size selection and sequenced using PacBio RS at Genome Quebec, Montreal, Canada (Table 1). No DNA shearing was performed on this sample since the DNA size distribution was close to the desired 12-15 kb size range.

**TABLE 1:** Accession numbers, sequencing information, and assembly statistics of metagenomes from four samples from Daisy the digester^*a*^

Sample ID	SRA, GenBank accession number	IMG taxon ID^*b*^	Reagent kit and sequencing platform	Number of read pairs	Assembly size (bp)^*c*^	Number of contigs	N50
S62W2	SRX17707942, JASEYM000000000.1	3300033757	Hiseq PE Cluster kit v4 cBot, Illumina Hi Seq 2500 PE	23,784,157	465,115,640	273,816	2,645
S80W1	SRX17707940, JASEYO000000000.1	3300033889	Hiseq PE Cluster kit v4 cBot, Illumina Hi Seq 2500 PE	24,589,275	442,686,435	370,202	1,283
S79W4	SRX17707941, JASEYN000000000.1	3300045909	NovaSeq 6000 S4 300cycles, Version 1.5, Illumina NovaSeq 6000	71,001,367	984,592,173	606,662	2,516
S80W4	SRX17707943	3300045909	Sequel II Sequencing Kit 2.0, PacBio Sequel II	5,589,701^*d*^	NA	NA	7,877^*e*^

^*a*^ GenBank accession numbers, assembly size, and N50 are for the SPAdes assembly of Illumina reads.

^*b*^ The IMG taxon id for S79W4 and S80W4 refers to the co-assembly of Illumina and PacBio reads, while the taxon id for S62W2 and S80W1 refers to the assembly of the Illumina reads.

^*c*^ Contigs > 500 bp before trimming were submitted to GenBank.

^*d*^ For PacBio this refers to the number of reads, not read pairs.

^*e*^ For PacBio this refers to the N50 for reads.

The Illumina reads from each sample were quality trimmed and assembled separately by the Anvi’o v 6.2 metagenomic Snakemake workflow (4). This pipeline uses Illumina-utilities (5) with the read quality control filtering developed by Minoche et al. (6), which involves B-tail trimming (low-quality bases at the end of reads), removal of reads containing uncalled bases, and keeping reads only if at least two-thirds of the bases of the first half of the read have quality values of *Q* ≥ 30. The quality-trimmed reads were assembled using SPAdes v. 3.12.0 (metaspades mode [7]). In addition, PacBio reads from S80W4 were combined with the Illumina reads from S79W4 in a hybrid assembly using SPAdes v3.14.1. Taxonomic profiling of the Illumina reads was done using phyloFlash 3.4, which assembles and classifies rRNA genes (8). Binning of the assemblies was done using metaBAT 2 v2.15 (9) and MaxBin 2 v.2.2.7 (10). Default parameters were used for all software. All bins generated from all assemblies were compared and dereplicated using dRep v.2.5.4 (11) with minimum completeness at 50%, maximum contamination at 25%, and average nucleotide identity threshold at 99%. The taxonomy of the resulting metagenome-assembled genomes (MAGs) was obtained by comparisons to the genome taxonomy database (12) using GTDB-tk 1.5.1 (13), while quality was assessed using Anvio v.7.1 and CheckM v.1.1.2 (14).

**Fig 1:**
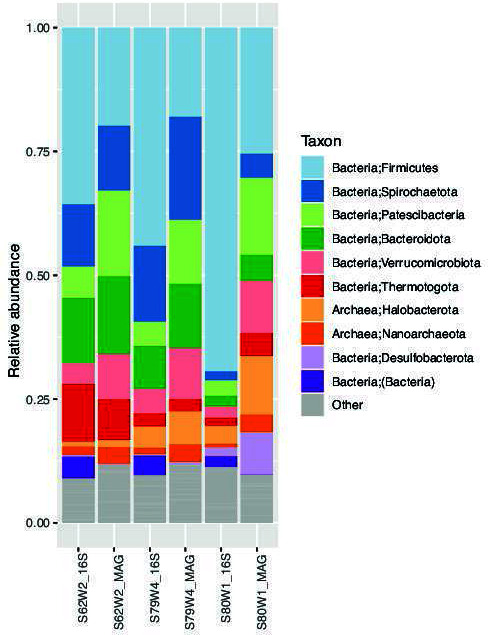
Relative abundance and taxonomic distribution of MAGs and rRNA genes obtained from the Illumina metagenome reads. The 10 most abundant phyla are shown. The 16S rRNA reads were obtained using the phyloflash v3.4 pipline (8) with classification to order level using the Ref NR99 database from SILVA v.138 (16). The abundance of MAGs was obtained from read mapping using the Anvi’o v.7.1 pipeline, and taxonomy was obtained from GTDB v. 202 and converted to SILVA v.138 format using the ar122_metadata_r202.xlsx and bac120_metadata_r202.xlsx provided for download at GTDB.

The sequencing information and assembly statistics are shown in Table 1. Binning and dereplication gave 281 MAGs, where 254 were of medium quality with completion > 50% and contamination < 10%, 166 of these had completeness ≥ 75% and contamination < 10%. The 10 most abundant phyla in the metagenomes based on rRNA genes and MAGs are shown in Fig. 1. Functional annotation of the metagenomes was done by JGI’s IMG database (15). These annotations will be used to understand the higher-than-expected efficiency of this solid-state digester configuration.

## Data Availability

The Illumina and PacBio reads, as well as spades assemblies of Illumina reads, are available at NCBI under BioProject PRJNA501900. See Table 1 for individual SRA and GenBank accession numbers. The assemblies are also available in IMG (15) under study ID Gs0130338. IMG taxon IDs are provided in Table 1. Quality statistics and taxonomy of each MAG (an Excel file) can be found on FigShare at https://doi.org/10.6084/m9.figshare.21071284. Fasta files of all the MAGs are provided at https://doi.org/10.6084/m9.figshare.20776180.v1.
